# Telestroke: Barriers to the Transition

**DOI:** 10.3389/fneur.2021.689191

**Published:** 2021-09-14

**Authors:** Chiara Busti, Alessio Gamboni, Giuseppe Calabrò, Mauro Zampolini, Marialuisa Zedde, Valeria Caso, Francesco Corea

**Affiliations:** ^1^Emergency Department, San Giovanni Battista Hospital of Foligno, Umbria, Italy; ^2^Stroke Unit, San Giovanni Battista Hospital of Foligno, Umbria, Italy; ^3^Neurology Unit, Stroke Unit, Azienda Unità Sanitaria Locale-IRCCS di Reggio Emilia, Reggio Emilia, Italy; ^4^Stroke Unit, Santa Maria Misericordia Hospital, Perugia, Italy

**Keywords:** stroke, barriers, telemedicine, telestroke, COVID-19

## Introduction

Only few patients affected by acute stroke are treated with thrombolytic therapy or endovascular treatment, especially in rural or underserved areas/hospitals. A survey of national scientific societies and stroke experts in 44 European countries conducted in 2019 showed that 7.3% of all patients with an ischemic stroke in Europe received intravenous thrombolysis (IVT), whereas 13 countries reported IVT rates of 10% or more (1). In rural areas, the problem is compounded by a general lack of stroke specialists with experience using tissue plasminogen activator on site. Telemedicine in stroke, or telestroke, has emerged as one of the most successful strategies to effectively treat acute ischemic stroke with IVT also in remote hospitals, providing the experience and competence of stroke teams to as many patients as possible (2–4). Remote evaluation *via* videoconference of the National Institute of Health Stroke Scale (NIHSS) has shown similar interrater reliability to on-site examination (5) and encouraging data reported the potential benefit of telestroke in identifying patients potentially eligible for endovascular treatment (6–8). At least home-based tele rehabilitation—defined as the use of telecommunication devices (telephone, videophone) by a clinician to offer evaluation and distance support for disabled persons living at home—is an excellent model to meet the rehabilitation needs of stroke survivors in resource-limited settings (9). There are many upfront costs involved with the initial installation of telestroke; training practitioners in its usage and reimbursement ambiguity creates limits, but despite this, telestroke appears cost-effective compared to usual care, as costs are upfront but benefits of improved stroke care are lifelong (10). Switzer et al. estimated that compared with no network, a telestroke system may result in more IVT, more endovascular stroke therapies, and more stroke patients discharged independently, and despite upfront and maintenance expenses, it results in greater cost savings for the entire network (11). An Acute Telestroke Programme commenced incrementally across Western Australia during 2016–2017. An economic evaluation study on effectiveness and cost-effectiveness of this program is, to date, ongoing (12).

The latest American Stroke Association guidelines for the early management of adults with ischemic stroke recommend the implementation of telemedicine in order to increase access to acute stroke care (13).

Despite the potential improvement of healthcare quality, also supported by guideline recommendations (14), the transition to larger telemedicine-based stroke networks remains marginal in Europe and Italy (15).

The 2020 COVID-19 pandemic boosted the implementation of Information and Communication Technology (ICT) resources in healthcare systems and in the setting of stroke. The approach of the healthcare authorities to the topic is general without any specific referral to telestroke, which lies inside the digital health issues. Some authors analyzed structural and nonstructural modifications of acute stroke care pathways undertaken at regional healthcare institutions in Italy after the COVID-19 pandemic, concluding that telemedicine is one of the best answers to approach stroke care in pandemic times (16). The implementation of the existing telestroke networks may minimize futile transfers; telestroke pathways starting at the patient's home could reduce unnecessary contact and reduce the risk of contagion; telemedicine at hospital level is crucial to evaluate patients suspected of having an acute neurological pathology in the COVID units or even in emergency departments.

International medical authorities in late December 2020 and at the beginning of 2021 released various statements and recommendations to normalize the framework of digital medicine for both device selection and regulatory measures. If barriers such as low reimbursement rates and high equipment costs will be reduced, telestroke has the potential to diminish the striking geographical disparities of acute stroke care.

Here we analyze the Food and Drug Administration (FDA), European Medicines Agency, and other national statements on this topic to address the best strategies and tactics to face this transition.

## Strategy for the Transition

National and international healthcare authorities depicted their “grand strategies” in various ways to encourage the deployment of digital health solutions. These are long-term plans that will be relevant for stroke clinicians in all settings, from primary care to hospital-based practice and rehabilitation.

The US healthcare authorities demonstrated a longstanding commitment to advancing regulatory science for digital health, already articulated in 2017 with the Digital Health Innovation Action Plan^1^. Later in 2020, the activity was strengthened with the creation of the Digital Health Center of Excellence (DHCoE), a specific office within the FDA.

The main mission of the DHCoE is to support the advance in science and evidence for digital health technologies that meet the needs of stakeholders; favor the access to highly specialized expertise, knowledge, and tools to accelerate access to digital health technology that maintain standards of safety and effectiveness; align regulatory approach to harmonize international regulatory expectations and industry standards; increase awareness and understanding of digital health trends; and reimagine medical device regulatory paradigm tailored for digital health technologies (17). These activities represent a powerful initiative to enhance the digital transition in the country. The US agencies have issued strong policies to support the uptake of these digital tools, especially during the recent public health emergency.

The European regulatory authorities in 2018 presented a broad list of policy priorities for the EU Commission plan for the years 2019–2024. This was built upon previous initiatives enhancing the creation of a Digital Single Market, starting from the assumption that the digital transition should benefit everyone, putting people first and opening new business opportunities. One of the seven flagship initiatives of the Europe 2020 strategy adopted by the Commission is the Digital Agenda, which has the diffusion of wide-band internet as a core point in many countries^2^. Health is one of the sectors included in this agenda, given the potential benefits that digital services could offer citizens and enterprises in this area. The main pillars are (1) to secure data access and sharing; (2) connecting and sharing data for research, diagnosis, and health; and (3) citizen empowerment for digital services. The EU approach is, as usual, a purely legal framework in which national authorities will have to decline specific approaches according to their sensitivity and strength (18).

The more advanced European legislation in the field is the *Digital Healthcare Act* (Digitale– Versorgung–Gesetz or DVG) promulgated in Germany. In the Digital Health Application (DIGA), we see how reimbursement is the key point for many digital health solutions, whose importance and value have been highlighted and expanded in the pandemic. The DIGA statement entitles all subjects covered by statutory health insurance to receive reimbursement for certain digital health applications. Devices are described according to their risk profile and their eminently digital technology. Also, monitoring purposes need to be stated and included in a register. After a formal check by the device insurer, a prescription by the physician is needed. The German digital act is probably the clearest framework available in the continent (19).

In 2018, Bernetti et al. conducted a study aimed at investigating the diffusion of telemedicine in Italian stroke networks. With an online questionnaire, they assessed the type of stroke care setting, volume of thrombolysis–thrombectomy/year per site, access to stroke care among different hospitals, the presence of image sharing protocols within the network or patient dispatchment screening, and types of network solutions. Their findings demonstrated that the use of telemedicine is widespread, but mostly with nonprofessional devices or nonmedical equipment (15). In Italy, as seen in Germany in late 2020, the national political authorities addressed the topic in the specific political offices. The *Conferenza Stato-Regioni* on December 17 subscribed an agreement on national indications for the delivery of digital healthcare and telemedicine. The document provides a framework for organizing a generic digital health integrated system without addressing specific technical issues or pathways. As Italy is not equipped with a national agency for Health Technology Assessment, this will presumably trigger a new fragmentation among the 20 regional healthcare authorities with variable approaches (20). In the more predictable scenario, local authorities will look at mainstream international market choices. In other cases, authorities will feel self-confident, preferring to develop local ICT solutions and accreditation with more uncertainty on deployment timing and expected results^3^.

## Tactics for the Transition

International indications and common sense encourage national and local healthcare providers to deploy digital health integrated solutions. Despite such a background, the transition for digital enriched stroke care still seems too far off.

Clinicians might adopt many tactics to foster the digital transition, also with patient associations and policymakers' support. The offer of time-saving and cost-cutting telemedicine solutions would be relevant in increasing efficiency of healthcare systems in times of resource scarcity (e.g., physician's shortage, ambulance transport, stroke selection for hospital dispatchment). Specific stakeholders, such as patient associations, could expect a rapid transition to telemedicine to get better home care for chronic patients, above all in times of pandemic (fragile stroke victims).

Also academic institutions may support this transition. The broader inclusion of digital health and telemedicine in medical school curricula may improve the training of doctors who are approaching telemedicine in their professional life, without any basic formal knowledge (21).

Scientific societies may put digital health and telemedicine in more central areas of interest (e.g., working groups, task forces, conferences) to ensure the highest possible quality of patient care. Diffusion of standard operating procedures (SOPs) in telestroke could help clinicians in the decision-making process. As in other emergency situations, it is crucial to standardize the procedures, for example, having a checklist of indications for or contraindications to IVT and thrombectomy, or a checklist to remember each step of the digital procedure. The medical community needs Continuing Medical Education on such topics, which appear indeed very dynamic (22).

Industry may be a relevant partner. In the world of pharmaceutical products and medical devices, mobile phone apps and/or web-based resources are already available and widespread. A teamwork methodology to synchronize the clinical activity with medical products would trigger transition, thus simplifying choices and avoiding redundancies (23).

Formalizing reimbursement strategies with governmental authorities or insurances is crucial for digital health transition. Both subjects may be strongly motivated to promote this change, as they are constantly interested in efficiency and savings (24). It is always crucial to guarantee the safety of healthcare by not being overwhelmed by insurance companies.

Telemedicine cannot substitute for standard clinical evaluation, but may guarantee a good standard of care also in geographically underserved areas, in the context of low resources or in times of pandemic^4^. Telemedicine consultations have shown to improve accuracy in decision making compared with telephone consultation and to increase safe local administration of IVT with similar results to patients presenting themselves directly at the tertiary stroke center. However, patients with moderate to severe ischemic stroke treated with thrombolysis may potentially benefit from care at a tertiary stroke center to receive the best care practice in etiology research, complication management, and rehabilitation. Hence, local thrombolysis administration does not completely mitigate the need for tertiary center care.

A primary analysis would suggest that medical unions may oppose health digitalization, considering it a failure of the conventional medical mission. On the other hand, digital health will support medical professionals in pursuing a modern healthcare model with higher standards and better safety profiles. Medical unions already demonstrated interest in considering salary benefits for healthcare providers delivering smart-working with telemedicine. The welfare countermeasures, such as smart-working options, may have some benefits for child care also for healthcare workers^5^.

The main stakeholders, the barriers reported, and potential benefits are summarized in Table 1.

**Table 1:** Telestroke: Stakeholder, barriers to the digital transition, potential countermeasures.

**Stakeholder**	**Barrier**	**Needs and potential benefits**
Medical school curricula	Lack training & education	Physicians and trainees need to acquire basic skills.
Medical societies	Lack of visibility for Telestroke and lack of SOPs^*^	The Scientific community needs to offer a governance to the digitalization of medicine. Supporting the transition with specific CME and offering visibility to careers investing in digital health.
Industry planning	No integration between Pharma industry and digital devices/ telemedicine.	Integrated Digital health will support the capacity of hospitals and the safety of medical treatment.
Patients associations	Customers perception	On the whole, citizens seem ready to use digital services for healthcare. This will save time and money.
Digitalization	Infrastructure outdated	Increase the accessibility for citizens to digital healthcare.
Insurance coverage	Lack of Reimbursement for the service from public or private insurers	Any procedure using digital devices needs to be recognized by authorities.
Medical Union	Resistance in the working market	Growth of potential activities and certainty of salary increase.

* *SOPs, standard operating procedures*.

## Conclusion

Although national political authorities pushed toward a digital health revolution, many barriers still stand. The strategies adopted were many, although not consistently effective. Evolved systems will enable the safe introduction of marketable solutions/devices with a medical prescription, but the standard evidence-based medicine approach may not be easily applicable. Therefore, we foster the paradigm introduced by Horwitz of a medicine-based evidence supported by digital health (25). The availability of digital health–integrated SOPs for main clinical lines would support the decision making process and reduce errors. Medical educational models should be reorganized on many levels, and stakeholders should reserve an open-minded approach to this process, in order to avoid the risk of being overwhelmed or miss useful opportunities.

## Author Contributions

All authors handled the draft and participated in the ideation of the paper.

## Conflict of Interest

The authors declare that the research was conducted in the absence of any commercial or financial relationships that could be construed as a potential conflict of interest.

## Publisher's Note

All claims expressed in this article are solely those of the authors and do not necessarily represent those of their affiliated organizations, or those of the publisher, the editors and the reviewers. Any product that may be evaluated in this article, or claim that may be made by its manufacturer, is not guaranteed or endorsed by the publisher.

## Footnotes

^1^Available online at: https://www.fda.gov/media/106331/download (accessed March 29, 2021).

^2^Available online at: https://www.europarl.europa.eu/factsheets/en/sheet/64/digital-agenda-for-europe (accessed March 17, 2021).

^3^Available online at: http://www.statoregioni.it/it/conferenza-stato-regioni/sedute-2020/seduta-del-17122020/atti/repertorio-atto-n-215csr/ (accessed March 17, 2021).

^4^Available online at: https://www.statnews.com/2020/06/25/telemedicine-time-to-shine-doctors-abandoning-it/

^5^Available online at: https://www.nysna.org/position-statement-telehealth-and-telemedicine#.YMGtOy98pQI
